# Ability of IMPROVE and IMPROVE-DD scores to predict outcomes in patients with severe COVID-19: a prospective observational study

**DOI:** 10.1038/s41598-022-17466-8

**Published:** 2022-08-03

**Authors:** Mina Adolf Helmy, Lydia Magdy Milad, Ahmed Hasanin, Yasmin S. Elbasha, Hala A. ElSabbagh, Mohamed S. Elmarzouky, Maha Mostafa, Amr K. Abdelhakeem, Mohamed Abd El-Monem Morsy

**Affiliations:** 1 Department of Anesthesia and Critical Care Medicine, Faculty of Medicine, Cairo University, Cairo, Egypt; 2 Department of General Surgery, Cairo University, Cairo, Egypt

**Keywords:** Biomarkers, Risk factors

## Abstract

In this study we aimed to evaluate the ability of IMPROVE and IMPROVE-DD scores in predicting in-hospital mortality in patients with severe COVID-19. This prospective observational study included adult patients with severe COVID-19 within 12 h from admission. We recorded patients' demographic and laboratory data, Charlson comorbidity index (CCI), SpO_2_ at room air, acute physiology and chronic health evaluation II (APACHE II), IMPROVE score and IMPROVE-DD score. In-hospital mortality and incidence of clinical worsening (the need for invasive mechanical ventilation, vasopressors, renal replacement therapy) were recorded. Our outcomes included the ability of the IMPROVE and IMPROVE-DD to predict in-hospital mortality and clinical worsening using the area under receiver operating characteristic curve (AUC) analysis. Multivariate analysis was used to detect independent risk factors for the study outcomes. Eighty-nine patients were available for the final analysis. The IMPROVE and IMPROVE-DD score showed the highest ability for predicting in-hospital mortality (AUC [95% confidence intervals {CI}] 0.96 [0.90–0.99] and 0.96 [0.90–0.99], respectively) in comparison to other risk stratification tools (APACHE II, CCI, SpO_2_). The AUC (95% CI) for IMPROVE and IMPROVE-DD to predict clinical worsening were 0.80 (0.70–0.88) and 0.79 (0.69–0.87), respectively. Using multivariate analysis, IMPROVE-DD and SpO_2_ were the only predictors for in-hospital mortality and clinical worsening. In patients with severe COVID-19, high IMPROVE and IMOROVE-DD scores showed excellent ability to predict in-hospital mortality and clinical worsening. Independent risk factors for in-hospital mortality and clinical worsening were IMPROVE-DD and SpO_2_.

## Introduction

Severe Coronavirus disease 2019 (COVID-19) is characterized by respiratory failure and severe inflammatory response as well as hypercoagulability. The hypercoagulability is manifested as micro- and macrovascular thrombosis and elevated D-dimer level^1^. The prevalence of venous thromboembolism (VTE) among patients with severe COVID-19 is higher than that in acutely ill surgical and nonsurgical patients admitted to the intensive care^2^, and is associated with higher risk of death^3^.

The International Medical Prevention Registry on Venous Thromboembolism (IMPROVE) and IMPROVE-D-dimer (IMPROVE-DD) scores are used to determine the risk of VTE in hospitalized, acutely ill medical patients^4^. These scores were derived from large international registry and included the independent risk factors for VTE (prior VTE, thrombophilia, paralysis of the lower extremity during the hospitalization, current malignancy, immobilization for at least 7 days, ICU admission, and age > 60 years)^4,5^ and each risk factor was given a weigh within the score according to its hazard ratio^4,5^. The IMPROVE score was then validated showing good ability to risk-stratify acutely ill medical patients for VTE^6^ and Similar findings had been reported in hospitalized patients with COVID-19^7^. In a retrospective analysis, high IMPROVE score was reported to be an independent predictor for 30-days mortality in patients with COVID-19 with undifferentiated severity^8^. To the best of our knowledge, there is no reports of prospectively evaluating the ability of IMPROVE and IMPROVE-DD score to predict outcomes in patients with severe COVID-19.

This study aims to evaluate the ability of IMPROVE and IMPROVE-DD scoring systems in predicting in-hospital mortality in patients with severe COVID-19.

## Methods

This prospective observational study was conducted in three separate intensive care units (ICUs) in Cairo University Hospital, after institutional Research Ethics Committee approval (N-104-2021) from November to December 2021. Written informed consent was obtained from the patient’s next-in-kin before the enrolment. We confirm that the research was performed in accordance with the Declaration of Helsinki. We included adult patients (> 18 years) with severe COVID-19 (confirmed to be positive for SARS-CoV-2 by the reverse-transcriptase polymerase chain reaction, peripheral oxygen saturation [SpO_2_] < 94% at room air, a respiratory rate > 30 breaths/min, arterial oxygen tension/fraction of inspired oxygen ratio [PaO_2_/FiO_2_] < 300 mm Hg, or lung infiltrates > 50% as detected by computed tomography [CT] of the chest). Patients who are expected to die or be discharged within 48 h after admission and pregnant women were excluded.

Within 12 h from the admission to the emergency department, patient’s demographic data (age, weight, height, sex, co-morbidities by calculating Charlson comorbidity index), clinical data (mean arterial pressure, heart rate, temperature, respiratory rate, SpO_2_ at room air, acute physiology and chronic health evaluation II [APACHE II] score), laboratory data (Hemoglobin, white blood cell count, platelet count, d-dimer, C-reactive protein), level of respiratory support (simple oxygen therapy, high-flow nasal oxygen, non-invasive ventilation, or invasive mechanical ventilation) were recorded. The risk scores for VTE including IMPROVE score (calculated as; prior VTE: 3 points, thrombophilia: 2 points, paralysis of the lower extremity during the hospitalization: 2 points, current malignancy: 2 points, immobilization for at least 7 days: 1 point, ICU admission: 1 point, age > 60 years: 1 point) and IMPROVE-DD score (as IMPROVE score in addition to giving 2 points if the D-dimer ≥ 2-time upper limit of normal) were calculated.

The patients were managed during the ICU stay according to our standardized protocol for respiratory and hemodynamic support^9–11^ and the treating physician was blinded to the purpose of the study. All patients were examined by venous color-Doppler ultrasound of the limbs to assess the presence of DVT upon ICU admission, by Samsung HS60 ultrasound machine with a high frequency linear probe. Subsequent investigation for VTE during the ICU stay was done upon suspicion in the form of venous color-Doppler ultrasound and multisliced CT angiography.

Patients with no documented VTE received prophylactic dose of low molecular weight heparin. If VTE was confirmed, the patient received therapeutic dose of low molecular weight heparin, the anticoagulant dose was adjusted if creatinine clearance < 30 mL/min. Only two patients did not receive any anticoagulation due to high bleeding risk and those patients did not develop VTE during their ICU course. All patients were followed up until discharge from the ICU or death.

Patient’s outcome data were recorded such as length of ICU stay, patient outcome (survival or death), incidence of VTE, Incidence of major bleeding, incidence of other complications, and incidence of clinical worsening (the need for conversion to more intense treatment: invasive mechanical ventilation, vasopressors, the need to renal replacement therapy).

The primary outcome was the accuracy of the IMPROVE and IMPROVE-DD scores to predict in-hospital mortality. Secondary outcomes included the accuracy of the IMPROVE, D-dimer, APACHE II, Charlson comorbidity index and SpO_2_ to predict in-hospital mortality. Accuracy of IMPROVE-DD, IMPROVE, D-dimer, APACHE II, Charlson comorbidity index and SpO_2_ to predict clinical worsening. Other outcomes included patient’s demographic data, clinical and laboratory data at admission.

### Sample size calculation

Sample size was calculated using MedCalc Software version 14 (MedCalc Software bvba, Ostend, Belgium) to detect area under receiver operating characteristic curve (AUC) of 0.75 with null hypothesis AUC of 0.5. Taking in consideration that the in-hospital mortality of severe COVID-19 is ⁓ 20%, we calculated a minimum number of 85 patients (with at least 17 deaths) for study power of 90% and alpha error of 0.05.

### Statistical analysis

The patients were categorized according to the survival (survived/dead) and clinical worsening (yes/no). Data were reported as mean and standard deviation or median and quartiles as appropriate and were analyzed using the unpaired student t-test or the Mann–Whitney test as appropriate. Categorical variables were summarized as frequency (percentages) and analyzed using the Chi-squared or Fisher’s exact test as appropriate. The AUC was calculated for IMPROVE and IMPROVE-DD scores as well as for the D-dimer, APACHE II, Charlson comorbidity index, and SpO_2_, to predict mortality and clinical worsening. The best cut-off values were calculated using the Youden’s index. The AUCs were compared using the Henley-MacNeil test. Logistic regression was performed to obtain adjusted odds ratio (OR) and 95% confidence intervals (CI) for mortality and clinical worsening. Statistical analysis was conducted using the MedCalc Software version 14 and Statistical package for social science (SPSS) software, version 26 for Microsoft Windows (Armonk, NY: IBM Corp).

## Results

**Figure 1:**
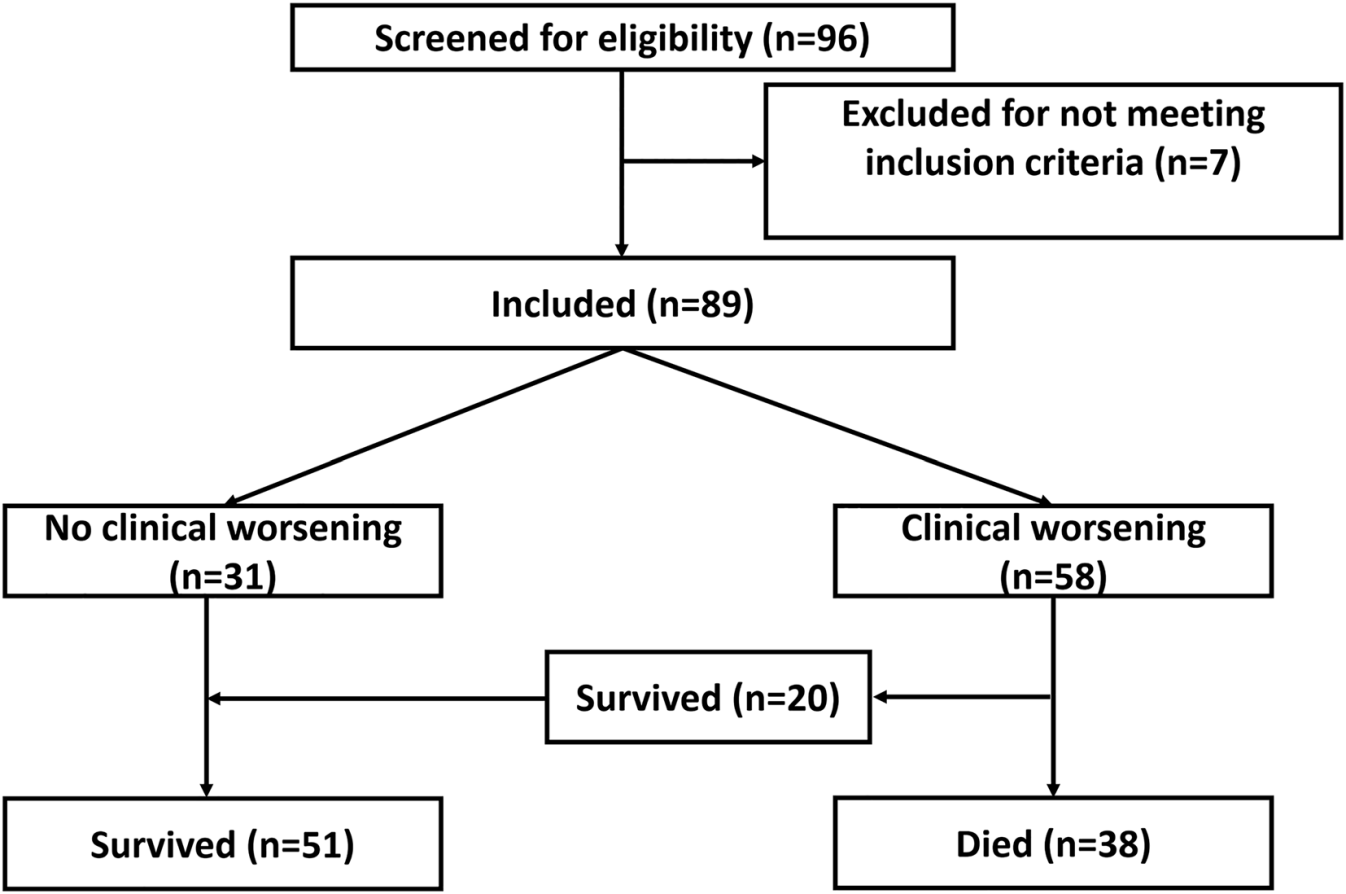
Patient’s enrolment.

Ninety-six patients were screened for eligibility, seven patients were excluded for not fulfilling the inclusions criteria, and 89 patients were included and were available for the final analysis. Clinical worsening occurred in 56 (63%) patients and 38 (43%) died (Fig. 1). On admission, all patients were on simple oxygen mask. During their ICU course, 52 (58%) patients required non-invasive respiratory support (high flow oxygen and/or non-invasive ventilation), 41 (46%) patients required invasive mechanical ventilation and only 2 (2%) patients were on ECMO.

The demographic, clinical, and laboratory data, and clinical course of the included patients according to the in-hospital mortality and clinical worsening are presented in Table 1 and Supplementary Table, respectively.

**Table 1:** Demographic data, clinical data, and ICU course.

	All (n = 89)	Survived (n = 51)	Died (n = 38)
Age (years), median (Q1, Q3)	66 (54, 72)	59 (50, 68)	70 (63, 74)
Male sex, n (%)	40 (45%)	25 (49%)	15 (40%)
BMI (kg), median (Q1, Q3)	28 (25, 31)	27 (25, 29)	29 (26, 32)
Heart rate (bpm), mean ± SD	96 ± 19	93 ± 17	100 ± 20
MAP (mmHg), mean ± SD	88 ± 15	88 ± 14	88 ± 16
RR (breath per minute), median (Q1, Q3)	25 (22, 32)	24 (20, 32)	28 (22, 34)
Temperature (°C), median (Q1, Q3)	37.5 (37.0, 38.2)	37.5 (37.0, 38.0)	38.0 (37.0, 38.5)
SpO_2_ (%), median (Q1, Q3)	84 (72, 89)	87 (80, 90)	76 (68, 85)
Symptoms to admission (days), median (Q1, Q3)	7.0 (4.5, 9.5)	7 (4, 10)	7 (5, 9)
APACHE II, median (Q1, Q3)	10 (6, 13)	8 (6, 11)	11 (9,15)
Charlson comorbidity index, median (Q1, Q3)	1 (0, 3)	1 (0, 2)	3 (1, 4)
IMPROVE score, median (Q1, Q3)	2 (1, 3)	2 (1, 2)	3 (3, 5)
IMPROVE-DD, median (Q1, Q3)	4 (2, 5)	2 (1, 3)	5 (5, 7)
D-dimer (μg/mL), median (Q1, Q3)	1.8 (0.6, 4.1)	0.7 (0.4, 1.8)	4.7 (2.2, 7.5)
CRP (mg/dL), median (Q1, Q3)	69 (28, 124)	56 (21, 100)	95 (46, 146)
Hemoglobin (gm/dL), median (Q1, Q3)	12 (10, 13)	12 (10, 13)	12 (9, 14)
White blood count (*10^12^/L), median (Q1, Q3)	9.2 (5.6, 14.6)	7.3 (4.8, 12)	9.9 (5.0, 15.2)
Platelets count (*10^3^/μL), median (Q1, Q3)	196 (150, 280)	235 (152, 303)	185 (135, 248)
INR, median (Q1, Q3)	1.1 (1.0, 1.2)	1.1 (1.0, 1.2)	1.1 (1.0, 1.3)
Anticoagulation, n (%)	87 (98%)	49 (96)	38 (100%)
Venous thromboembolism, n (%)	7 (8%)	0 (0%)	7 (18%)
Major bleeding, n (%)	12 (14%)	5 (10%)	7 (18%)
Vasopressors, n (%)	41 (46%)	3 (6%)	38 (100%)
Sepsis, n (%)	24 (27%)	2 (4%)	22 (58%)
Renal replacement therapy, n (%)	17 (19%)	5 (10%)	12 (32%)
Clinical worsening, n (%)	56 (65%)	18 (35%)	38 (100%)
Days to worsening, n (%)	2 (1, 4)	3 (1, 3)	2 (1, 4)
Other complications, n (%)	19 (21%)	5 (10%)	14 (37%)
ICU stay (days), n (%)	10 (8, 16)	10 (8, 15)	11 (7, 17)

*APACHE II* acute physiology and chronic health evaluation II, *BMI* body mass index, *CI* confidence interval, *CRP* C-reactive protein, *DD* D-dimer, *ICU* intensive care unit, *IMPROVE* international medical prevention registry on venous thromboembolism, *INR* international normalized ratio, *MAP* mean arterial pressure, *RR* respiratory rate, *SD* standard deviation, *SpO*_*2*_ peripheral oxygen saturation, *Q *quartiles.

**Figure 2:**
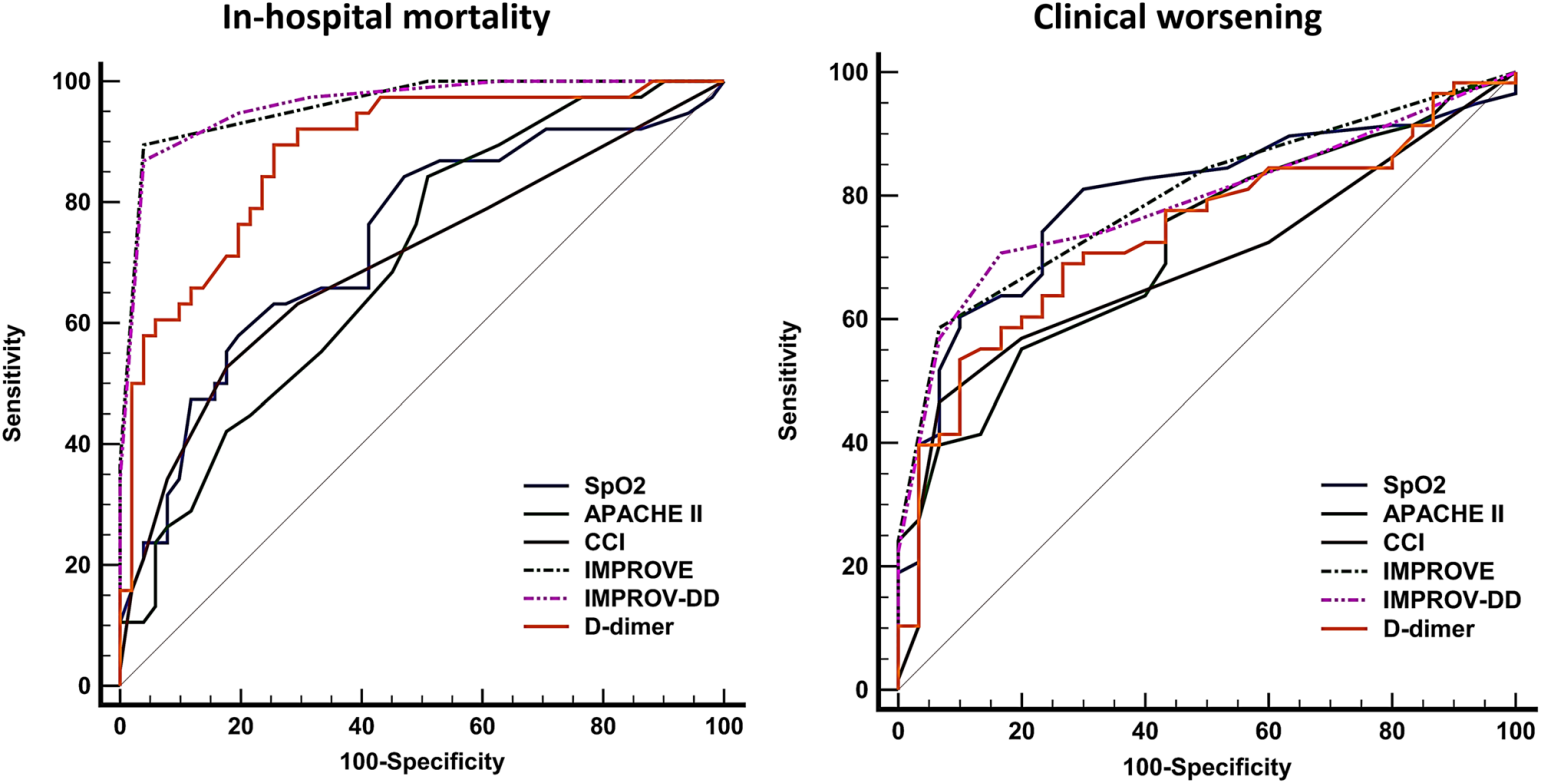
AUC analysis for the ability to predict in-hospital mortality (left) and clinical worsening (right). *APACHE II* acute physiology and chronic health evaluation II, *CCI* Charlson comorbidity index, *DD* D-dimer, *IMPROVE* international medical prevention registry on venous thromboembolism, *SpO*_*2*_ peripheral oxygen saturation.

The IMPROVE and IMPROVE-DD score showed the highest AUC (AUC [95% CI] 0.96 [0.90–0.99] and 0.96 [0.90–0.99], respectively) for predicting in-hospital mortality in comparison to the SpO_2_, APACHE II score, Charlson comorbidity index and D-dimer (Table 2, Fig. 2).

**Table 2:** The AUC analysis for prediction of in-hospital mortality and clinical worsening.

	AUC (95%CI)	Sensitivity % (95% CI)	Specificity % (95% CI)	PPV % (95% CI)	NPV % (95% CI)	Cut-off value
**In-hospital mortality**						
IMPROVE^‡^	0.96 (0.90–0.99)	89 (75–97)	96 (87–100)	94 (81–99)	93 (82–98)	> 2
IMPROVE-DD^‡^	0.96 (0.90–0.99)	87 (72–96)	96 (87–100)	94 (80–99)	91 (80–97)	> 4
D-dimer	0.88 (0.80–0.94)	89 (75–97)	75 (60–86)	72 (57–84)	91 (77–97)	> 1.3 μg/mL
APACHE II*^†‡^	0.70 (0.59–0.79)	84 (69–94)	49 (35–63)	55 (42–68)	81 (63–93)	> 7
Charlson comorbidity index*^†‡^	0.70 (0.59–0.79)	53 (36–69)	82 (69–92)	69 (49–85)	70 (57–81)	> 2
SpO_2_*^†^	0.74 (0.63–0.82)	58 (41–74)	80 (67–90)	69 (50–84)	72 (59–83)	≤ 79%
**Clinical worsening**						
IMPROVE	0.80 (0.70–0.88)	60 (47–74)	94 (80–99)	94 (81–99)	59 (44–72)	> 2
IMPROVE-DD	0.79 (0.69–0.87)	71 (58–83)	82 (65–93)	87 (74–95)	63 (47–77)	> 3
D-dimer	0.74 (0.64–0.83)	70 (56–81)	73 (55–87)	81 (67–91)	59 (42–74)	> 1.27 μg/mL
APACHE II	0.71 (0.60–0.80)	55 (42–68)	80 (61–92)	84 (69–94)	48 (34–63)	> 10
Charlson comorbidity index*	0.68 (0.57–0.78)	46 (33–60)	91 (76–98)	90 (73–98)	50 (37–63)	> 2
SpO_2_	0.79 (0.70–0.87)	63 (49–75)	91 (76–98)	92 (79–98)	59 (44–72)	≤ 81%

*APACHE II* acute physiology and chronic health evaluation II, *AUC* area under receiver operating characteristic curve, *CI* confidence interval, *DD* D-dimer, *IMPROVE* international medical prevention registry on venous thromboembolism, *NPV* negative predictive value, *PPV* positive predictive value, *SpO*_*2*_ peripheral oxygen saturation.

*Denotes significance in relation to the IMPROVE.

^†^Denotes significance in relation to the IMPROVE-DD.

^‡^Denotes significance in relation to the D-dimer.

For the ability to predict clinical worsening, the IMPROVE score had the highest AUC (0.80 [0.70–0.88]) but was only significant when compared to the Charlson comorbidity index, *P* value: 0.049 (Table 2, Fig. 2). Furthermore, the IMPROVE score showed the highest specificity (94%) for predicting clinical worsening (Table 2, Fig. 2).

In the univariate analysis, risk factors for in-hospital mortality were the age, SpO_2_, APACHE II score, Charlson comorbidity index, IMPROVE score, IMPROVE-DD score, D-dimer, and C-reactive protein. The risk factors for clinical worsening were the respiratory rate, SpO_2_, APACHE II score, Charlson comorbidity index, IMPROVE score, IMPROVE-DD score, D-dimer, and C-reactive protein (Table 3).

**Table 3:** Univariate analysis for in-hospital mortality and clinical worsening.

	Mortality (n = 38)		Clinical worsening (n = 56)	
	Odd ratio (95% CI)	*P* value	Odd ratio (95% CI)	*P* value
Age (years)	1.04 (1.01–1.10)	0.014*	1.02 (0.99–1.05)	0.125
Male sex	0.68 (0.19–1.59)	0.371	1.18 (0.49–2.80)	0.714
BMI (kg)	1.03 (0.96–1.10)	0.472	1.05 (0.97–1.13)	0.249
Heart rate (bpm)	1.02 (1.00–1.05)	0.060	1.02 (1.00–1.05)	0.056
MAP (mmHg)	1.00 (0.97–1.03)	0.940	1.01 (0.98–1.04)	0.719
RR (breath per minute)	1.06 (0.99–1.13)	0.089	1.11 (1.03–1.20)	0.005*
Temperature (°C)	1.84 (0.98–3.46)	0.058	1.04 (0.56–1.95)	0.897
SpO_2_ (%)	0.92 (0.87–0.96)	< 0.001*	0.87 (0.80–0.93)	< 0.001*
Symptoms to admission (days)	1.05 (0.95–1.16)	0.381	0.98 (0.89–1.01)	0.740
APACHE II	1.16 (1.06–1.28)	0.002*	1.18 (1.06–1.32)	0.003*
Charlson comorbidity index	1.57 (1.21–2.04)	0.001*	1.57 (1.16–2.13)	0.004*
IMPROVE score	64.72 (11.39–367.65)	< 0.001*	3.73 (1.96–7.10)	< 0.001*
IMPROVE-DD	6.73 (2.87–15.71)	< 0.001*	1.93 (1.43–2.62)	< 0.001*
D-dimer (μg/mL)	1.87 (1.38–2.53)	< 0.001*	1.45 (1.13–1.87)	0.004*
CRP (mg/dL)	1.01 (1.00–1.01)	0.026*	1.01 (1.00–1.02)	0.015*
Hemoglobin (gm/dL)	0.91 (0.75–1.10)	0.307	0.93 (0.77–1.13)	0.453
White blood count (*10^12^/L)	1.04 (0.97–1.12)	0.248	1.06 (0.98–1.15)	0.136
Platelet’s count (*10^3^/μL)	1.00 (0.99–1.00)	0.052	1.00 (0.99–1.00)	0.230
INR	1.28 (0.38–4.25)	0.693	16.41 (0.63–429.93)	0.093
Venous thromboembolism	24.53 (1.11–540.61)	0.999	8.89 (0.40–195.96)	0.999

*APACHE II* acute physiology and chronic health evaluation II, *BMI* body mass index, *CI* confidence interval, *CRP* C-reactive protein, *DD* D-dimer, *IMPROVE* international medical prevention registry on venous thromboembolism, *INR* international normalized ratio, *MAP* mean arterial pressure, *RR* respiratory rate, *SpO*_*2*_ peripheral oxygen saturation.

In the multivariate analysis we only included the APACHE II (instead of the age in the in-hospital mortality model or the respiratory rate in the clinical worsening model), IMPROVE-DD (instead of the IMPROVE and D-dimer in both model) to avoid collinearity; in addition to the Charlson comorbidity index, and C-reactive protein. Only the SpO_2_ and IMPROVE-DD were found to be the independent risk factors for in-hospital mortality (OR [95% CI] 0.85 [0.76–0.96] and 7.48 [2.36–23.66], respectively) and clinical worsening (OR [95% CI] 0.83 [0.75–0.92] and 1.55 [1.07–2.24], respectively) (Table 4).

**Table 4:** Multivariate analysis for in-hospital mortality and clinical worsening.

	Mortality (n = 38)		Clinical worsening (n = 56)	
	Odd ratio (95% CI)	*P* value	Odd ratio (95% CI)	*P* value
SpO_2_ (%)	0.85 (0.76–0.96)	0.010	0.84 (0.76–0.92)	< 0.001
APACHE II	1.03 (0.85–1.26)	0.766	1.14 (0.96–1.35)	0.142
Charlson comorbidity index	1.27 (0.69–2.34)	0.443	1.50 (0.90–2.41)	0.128
IMPROVE-DD	7.48 (2.36–23.66)	0.001	1.56 (1.09–2.23)	0.015
CRP (mg/dL)	1.01 (1.00–1.02)	0.208	1.01 (1.00–1.02)	0.063

*APACHE II* acute physiology and chronic health evaluation II, *CI* confidence interval, *CRP* C-reactive protein, *DD* D-dimer, *IMPROVE* international medical prevention registry on venous thromboembolism, *SpO*_*2*_ peripheral oxygen saturation.

## Discussion

In our cohort of patients with severe COVID-19, IMPROVE-DD score and SpO_2_, measured on hospital admission, were the two independent risk factors for clinical worsening and in-hospital mortality. IMPROVE-DD score showed excellent ability to predict in-hospital mortality which was the highest among all other tools for risk stratification. Furthermore, IMPROVE-DD score showed very good ability to predict clinical worsening especially in the positive predictive value. The IMPROVE and IMPROVE-DD scores showed an important and unique feature among all risk stratification tools which is combination of excellent positive and negative predictive values which was > 90% in the two scores.

The evidence for the association of COVID-19 with pro-coagulant state is well established despite the lack of definitive explanation^2^. IMPROVE-DD score had been originally introduced for estimation of the risk of VTE and to guide the prescription of anticoagulant drugs among hospitalized patients^4^. Furthermore, high VTE risk (using another VTE risk score: the Padua prediction score) was associated with increased risk of mortality in non-COVID acutely ill medical patients^12,13^. Therefore, we hypothesized that this score might show good performance in risk stratification of COVID-19. We found that IMPROVE-DD score was independently associated with risk of mortality even when clinical VTE was included in the analysis; this finding denotes that COVID-19 is associated with thrombotic complications which are not always clinically detected and are sometimes microvascular. This explains the relation between IMPROVE-DD score and patient outcomes despite the low incidence of VTE (8%) in our patients.

The APACHE II and Charlson comorbidity index are known risk stratification tools and were able to predict mortality in patients with COVID-19^14,15^. In this study, the predictive values of IMPROVE-DD score were higher than the APACHE II score and Charlson comorbidity index. Furthermore, when these scores were included in the multivariate analysis, the IMPROVE-DD score was found to be an independent predictor for poor outcome. The SpO_2_ was the other independent risk factor for poor outcomes in this study, this finding agreed with our previous reports^16,17^. Furthermore, the cutoff value for SpO_2_ (78%) for in-hospital mortality was, interestingly, close to our previous report (79%)^16^. We evaluated the IMPROVE-DD score to find whether incorporation of the D-dimer results would increase the validity of the score; however, the predictive value did not differ in the two scores.

One previous report by Greco et al.^8^, showed that the IMPROVE score was not an independent predictor of the need for intensification of treatment; however, it was a risk factor for mortality. The unexplained contradiction in the results of Greco et al., might be due to the retrospective design, the small sample (51 patients), and the lack of evaluation of the severity of illness in the included patients. Our study had the advantage of the larger sample size, the prospective design, the strict inclusion of severe cases, and the follow-up of patients until death or discharge. Another advantage is including Charlson comorbidity index in the multivariate analysis as an indicator of general status and chronic illness and this provide more accurate estimation for the validity of risk factors.

Currently, the ideal anticoagulation regimen for non-critically ill patients with COVID-19 is controversial^1^. The guidelines for prescription of anticoagulant drugs changed many times since the beginning of this pandemic. The latest evidence suggests that the benefit of therapeutic anticoagulation is clearer in the early stages of the disease, namely in non-critically ill, while prophylactic anticoagulation is more suitable in critically ill patients^18,19^. However, higher levels of anticoagulation increase the risk of bleeding which is sometimes serious. Hence, it is essential to select the level of anticoagulation meticulously and to find more tools which can guide this critical decision. Our findings suggest that patients with severe COVID-19 and IMPROVE score > 2 or IMPROVE-DD score > 3 have high risk of clinical worsening with positive predictive values of 94% and 87%, respectively. An IMPROVE score > 2 and IMPROVE-DD score > 4 have positive predictive value for in-hospital mortality of 94%. Our findings would improve the process of triaging and early detection of critical patients; furthermore, our findings might guide the decision regarding the level of anticoagulation in non-critically ill patients with COVID-19 such as providing more liberal anti-coagulation in high-risk patients and being more conservative in low-risk patients especially if they have high bleeding risk. Future studies are needed to evaluate the usefulness of incorporating those scores in clinical decision regarding the reduction of risk of clinical worsening and mortality.

Our study has a limitation for being conducted in one university, however, it has the advantage of being conducted in three separate ICUs which are completely different in their location (in different hospitals) and staff members. This provides our findings more generalizability. In the current study, the optimum cut-off value and subsequently the corresponding predictive values were derived from the AUC analysis of the included cohort; future studies are needed to validate these values.

## Conclusion

In patients with severe COVID-19, IMPROVE and IMPROVE-DD scores showed excellent ability to predict in-hospital mortality and clinical worsening. The two scores predicted in-hospital mortality with a PPV of 94% and a NPV 93% and 91%, respectively. The IMPROVE-DD score and SpO_2_ were independent risk factors for clinical worsening and in-hospital mortality.

## Supplementary Information

Supplementary Tables.

## Data Availability

The datasets used and/or analyzed during the current study are available from the corresponding author on reasonable request.
