# Supplementary material for: Ability of IMPROVE and IMPROVE-DD scores to predict outcomes in patients with severe COVID-19: a prospective observational study
Source: Sci Rep. 2022 Aug 3;12:13323. doi: 10.1038/s41598-022-17466-8 (PMC9349222; doi:10.1038/s41598-022-17466-8)
Supplement: Supplementary file 1 — Supplementary Tables. [file 41598_2022_17466_MOESM1_ESM.docx]

**Ability of IMPROVE and IMPROVE-DD scores to predict outcomes in patients with severe COVID-19: a prospective observational study**

Mina Adolf Helmy (MD) ^a^, Lydia Magdy Milad (MBBCh.) ^a^, Ahmed Hasanin (MD, DESA) ^a*^, Mohamed Abd El-Monem Morsy (MD) ^a^, Yasmin S Elbasha (MSc) ^a^, Hala A ElSabbagh (MSc)^a^, Mohamed S Elmarzouky (MD)^b^, Maha Mostafa (MD) ^a^, Amr K Abdelhakim (MD)^a^

^a^ Department of Anesthesia and Critical Care Medicine, Cairo University, Cairo, Egypt.

^b^ Department of general surgery, Cairo University, Cairo, Egypt.

**Supplementary table: Demographic data, clinical data, and ICU course according to the clinical course status. Data presented as mean ±standard deviation, median (quartiles), and frequency (%)**

|  | No clinical worsening (n= 33) | Clinical worsening (n= 56) |
| --- | --- | --- |
| Age (years) | 59 (51, 67) | 69 (57, 74) |
| Male sex | 14 (42%) | 26 (46%) |
| BMI (kg) | 28 (25, 31) | 29 (26, 31) |
| Heart rate (bpm) | 91 ±19 | 99 ±18 |
| MAP (mmHg) | 86 ±13 | 89 ±16 |
| RR (breath per minute) | 24 (18, 28) | 28 (22, 35) |
| Temperature (˚C) | 37.5 (37.2, 38.2) | 37.5 (37.0, 38.4) |
| SpO_2_ (%) | 88 (86, 90) | 79 (70, 86) |
| Symptoms to admission (days) | 7 (4, 10) | 7 (5, 9) |
| APACHE II | 7 (6, 10) | 11 (8, 14) |
| CCI | 1 (0, 1) | 2 (0, 4) |
| IMPROVE score | 2 (1, 2) | 3 (2, 4) |
| IMPROVE-DD | 2 (1, 3) | 5 (3, 5) |
| D-dimer (μg/mL) | 0.7 (0.4, 1.8) | 3.0 (0.9, 5.6) |
| CRP (mg/dL) | 47 (20, 73) | 90 (46, 133) |
| Hemoglobin (gm/dL) | 11.8 (10.3, 13.0) | 11.9 (9.9, 13.4) |
| White blood count (*10^12^/L) | 7.5 (5.4, 10.7) | 9.5 (5.9, 14.9) |
| Platelet count (*10^3^/μL) | 189 (153, 307) | 197 (141, 273) |
| INR | 1.1 (1.0, 1.2) | 1.1 (1.0, 1.3) |
| Venous thromboembolism | 0 (0%) | 7 (13%) |
| Major Bleeding | 2 (6%) | 10 (18%) |
| Other Complications | 4 (12%) | 15 (27%) |
| ICU stay (days) | 9 (8, 11) | 13 (9, 18) |

APACHE II: Acute Physiology and Chronic Health Evaluation II, BMI: body mass index, CCI: Charlson Comorbidity Index, CI: confidence interval, CRP: C-reactive protein, DD: D-dimer, ICU: intensive care unit, IMPROVE: International Medical Prevention Registry on Venous Thromboembolism, INR: international normalized ratio, MAP: mean arterial pressure, RR: respiratory rate, SpO_2_: peripheral oxygen saturation.
